# Insurance mechanisms for tropical cyclones and droughts in Pacific Small Island Developing States

**DOI:** 10.4102/jamba.v8i1.288

**Published:** 2016-08-31

**Authors:** Florent Baarsch, Ilan Kelman

**Affiliations:** 1 Climate Analytics gGmbH, Berlin, Germany; 2 Potsdam Institute for Climate Impact Research, Potsdam, Germany; 3 CESifo, Munich, Germany; 4 Institute for Risk and Disaster Reduction and Institute for Global Health, University College London, United Kingdom; 5 University of Agder, Kristiansand, Norway

## Abstract

One group of locations significantly affected by climate-related losses and damage is the Small Island Developing States (SIDS). One mechanism aiming to reduce such adverse impacts is insurance, with a wide variety of products and models available. Insurance for climate-related hazards affecting Pacific SIDS has not been investigated in detail. This article contributes to filling this gap by exploring how insurance mechanisms might be implemented in the Pacific SIDS for tropical cyclones and droughts. The study examines opportunities and constraints or limitations of some existing insurance mechanisms and programmes as applied to the Pacific SIDS. Eight insurance mechanisms are compared and discussed regarding the premium cost compared to the gross domestic product per capita, the amount of payout compared to the damage cost, the reserve and reinsurance, and the disaster risk reduction incentives. As such, this article offers a decision-making tool on insurance development for the Pacific SIDS. Ultimately, implementing disaster insurance for the Pacific SIDS depends on political will and external technical and financial assistance.

## Introduction

One group of countries experiencing losses and damage connected to tropical cyclones and droughts is the Small Island Developing States (SIDS), a United Nations grouping comprising of low-lying island and coastal countries sharing similar sustainable development challenges. One mechanism aiming to reduce such adverse impacts is insurance, with a wide variety of products and models available. Insurance for climate-related hazards affecting Pacific SIDS has not been investigated in detail, despite the frequency and intensity with which tropical cyclones and droughts affect many of the communities. A gap remains in understanding the usefulness and utility of insurance mechanisms for Pacific SIDS in dealing with these hazards. This article explores how insurance mechanisms might be implemented in the Pacific SIDS for tropical cyclones and droughts, examining opportunities and constraints or limitations of some existing insurance mechanisms and programmes as applied to the Pacific SIDS.

The Pacific SIDS countries considered are American Samoa, Cook Islands, Federated States of Micronesia, Fiji, French Polynesia, Guam, Kiribati, Marshall Islands, Northern Mariana Islands, Nauru, New Caledonia, Niue, Palau, Papua New Guinea (PNG), Samoa, Solomon Islands, Tonga, Tuvalu and Vanuatu. There exist many differences in culture, topography, population numbers, environment, governance forms and livelihoods, amongst others. In terms of similarities, scarce groundwater resources, many low-lying communities, lack of livelihood diversity and frequent dependence on outside support in many forms contribute to the impacts of droughts and tropical cyclones in Pacific SIDS communities.

To investigate possible contributions from insurance for dealing with or preventing losses and damage, the second part of this article describes how the Pacific SIDS are affected by tropical cyclones and droughts. This description defines the range of financial means needed for disaster compensation to set the stage for comparing eight existing insurance mechanisms for the Pacific SIDS. The last part of this study analyses, evaluates and discusses the insurance approaches for the Pacific SIDS in order to provide recommendations.

## Tropical cyclones and droughts in the Pacific Small Island Developing States

For Pacific SIDS:

a tropical cyclone is defined as a non-frontal low pressure system of synoptic scale developing over warm waters having organised convection and a maximum mean wind speed of 34 knots or greater extending more than half-way around near the centre and persisting for at least six hours. (Australian Bureau of Meteorology 2015:n.p.)

Each tropical cyclone usually affects only 1–3 Pacific SIDS, although Cyclone Ofa in 1990 hit Tuvalu, Tokelau, Tonga, Western Samoa, American Samoa and Niue (UNDHA 1990). The determination of accurate and precise losses and damage from a particular storm is subject to large uncertainties; however, between 1900 and 2006, the estimated average economic cost of a tropical cyclone striking Pacific SIDS was $75.7 million, ranging from $6m to $319.31m (World Bank 2006). Any insurance mechanism must cope with this large range.

The term ‘drought’ has a more complicated definition than ‘tropical cyclone’. Four main types of drought tend to be defined (American Meteorological Society 1997) – meteorological, agricultural, hydrological and socio-economic – although definitional debates regarding drought are long-standing (Glantz & Katz 1977; Heim 2002). Here, ‘drought’ focuses on precipitation and water available for use, that is, considering meteorological, agricultural and hydrological droughts (White, Falkland & Scott 1999).

Meteorological drought is defined as ‘an interval of time during which the supply of moisture at a given place cumulatively falls below the climatologically appropriate moisture supply’ (White *et al*. 1999:6). Because Pacific SIDS are very small in terms of land area and generally do not have either snowfall or glaciers, meteorological and hydrological droughts usually overlap as water resources come from rainfall. Agricultural drought is defined as ‘an interval of time when soil moisture cannot meet the evapotranspiration demand for crop initiation, to sustain crops and pastures or supply water for livestock or irrigated crops’ (White *et al*. 1999:6). The droughts affect freshwater supplies needed for human consumption (e.g. drinking and washing), irrigating crops and livestock production.

Over the last 30 years, drought costs in the Pacific SIDS are estimated to be between $0.24m and $124.74m per event (SOPAC 2005). As with cyclones, an insurance mechanism needs to be able to cope with this large range of impacts and losses.

### International post-disaster aid: The main disaster insurance?

Although significant indigenous capabilities exist amongst Pacific SIDS to prevent and deal with disasters, financial and technical support for short-term emergency needs and for longer-term reconstruction is still frequently requested and provided (see Boyle 1992; Kelman *et al*. 2011; Lewis 1999; Reardon 1992). For example, in the aftermath of Cyclone Tomas in 2010, at least eight different countries, four international non-governmental groups, two United Nations (UN) agencies and one private company provided assistance to Fiji (ADRA Australia 2010; French Embassy in Australia 2011; Government of Fiji 2010).

The assistance mentioned above has some limitations: the amount, effectiveness and timeliness of post-disaster assistance cannot be controlled by the location affected (Ferris 2011). Grants can lead to aid dependency while loans can lead to a financial burden. For example, the World Bank proposes assistance for reconstruction through grants and concessional loans. Even though the loans offered have a low interest rate and a long repayment period, they increase indebtedness. After the 2009 tsunami in Samoa, the World Bank approved financing of $11.8m of which $10m was a concessional loan (World Bank 2010b) adding approximately 5% onto the country’s external debt. Regarding aid dependency, after Cyclone Heta struck Niue in 2004, the Government of New Zealand provided NZ$20m to reconstruct the destroyed hospital (ReliefWeb 2004). This is comparable to the country’s 2004 gross domestic product (GDP) of NZ$17.71m (SPC 2009).

Aid dependency can also be disadvantageous when aid pledges are not followed through. After many disasters, donors deliver only 10% – 20% of the aid amounts that they initially pledged (Ferris 2011). Rather than reconstructing to withstand the next major hazard, unfulfilled pledges can lead to a cycle of vulnerability and disaster. Instead of assuming that aid (effectively insurance via charity) or loans will inevitably be available, other forms of insurance could contribute to dealing with future disaster losses and damage, possibly permitting a SIDS to better control its own disaster-related financing.

### Common insurance despite diversity?

Given the similarities and differences amongst the Pacific SIDS, are common insurance mechanisms viable? Although the SIDS group is based on their similarities, they have diverse economies and demographics. Populations range from just over 1000 in Niue to 6.3m in PNG which, in fact, has a population more than twice the sum of the population of all other Pacific SIDS. GDPs per capita span an order of magnitude (World Bank 2015). Despite these disparities, the Pacific SIDS have two main insurance-relevant strengths.

Firstly, the small land area and small populations assist in implementing projects. After Cyclones Ofa (1990) and Val (1991) hit Samoa, together killing almost two dozen people and causing about $500m of damage, the World Bank and the Government of Samoa initiated a disaster risk reduction (DRR) strategy in 2004 (World Bank 2004). Although this project had a budget of just $6.05m (about $33 per capita over the 4 years), it addressed the entire country (World Bank 2009). Similarly, when coastal management plans were developed for Samoa’s DRR, starting at the local level and integrating them at the district and national levels, plans were developed for the entire country’s coastline in 7 years (Daly *et al*. 2010). The small scale of Pacific SIDS (except PNG) could ease implementation of any insurance mechanism. Conversely, a disadvantage of small scale is that it makes pooling resources more difficult.

Secondly, a long history of regional cooperation, despite the distances between the Pacific SIDS is another strength. The Pacific has several regional organisations assisting with DRR, including activities relevant to climate change, the main ones being the Secretariat of the Pacific Community’s Applied Geoscience and Technology Division (SPC SOPAC) and the Secretariat of the Pacific Regional Environment Programme (SPREP). These regional organisations pool resources and capabilities for addressing topics for which each individual SIDS might not have the capability (Tutangata and Power 2002). The technical skills within these regional organisations can assist in developing a region-wide approach while allowing the countries to share investments or establishment costs for capital-intensive activities.

## Comparing insurance mechanisms for Pacific Small Island Developing States

This study selects eight insurance mechanisms over seven main insurance categories from the literature (e.g. Crichton 2008; Linnerooth-Bayer & Mechler 2006) to consider the relevance and applicability to the Pacific SIDS:

1. Multi-country pool insurance, with an example being the Caribbean Catastrophe Risk Insurance Facility (CCRIF) (CCRIF 2010; McGee, Phelan & Wenta 2014; UNECLAC 2010; World Bank 2008).
2. Catastrophe-linked securities, with an example being the Mexico Catastrophe Bond (MCB) (GFDRR & World Bank 2011b; Michel-Kerjan *et al*. 2011).
3. A compulsory public–private insurance mechanism, with an example being the Turkish Catastrophe Insurance Pool (TCIP) (GFDRR & World Bank 2011a; TCIP 2011).
4. International post-disaster assistance (Ferris 2011), including remittances (Le De *et al*. 2015), effectively acting as informal or ad hoc insurance.
5. Risk retention, implemented by many poor families because they cannot afford to invest in advance; in effect, self-insurance.
6. Private individual policies, for instance, sold by the company QBE in Vanuatu (QBE Insurance Limited 2011).
7a. Crop and weather micro-insurance, for example, Rashtriya Krishi Bima Yojana (RKBY) in India (Agriculture Insurance Company of India Limited 2010; Government of India c. 2014).
7b. A social safety net programme for small-scale farmers against the consequences of weather events, the Horn of Africa Risk Transfer for Adaptation (HARITA) in Ethiopia (Osgood 2010).

The first four are generally public insurance mechanisms; the latter four mechanisms are generally private, although the categories are not always delineated perfectly. Based on the literature on these mechanisms, as cited in the list above, the criteria used to compare their suitability for Pacific SIDS are as follows:

- GDP per capita.
- Percentage of grants from Official Development Assistance (ODA) in government revenues.
- Level of government indebtedness.

### Appraisal criteria

Based on the literature above, six main criteria to appraise insurance mechanisms are considered:

- The cost of premiums, its calculation method and the purchaser’s cost, to assess affordability and access. All premiums reported here are annual.
- The amount of payout – namely, the nature, sectors and percentage of the sum insured – to assess post-disaster client compensation expectations.
- The reserve or reinsurance to assess the stability and reliability (Cummins & Trainar 2009).
- DRR incentives as part of the insurance to assess loss reduction approaches (Crichton 2008).
- The contractual security of the mechanism to determine whether the beneficiary is entitled to receive a payout or support.
- The scope of coverage to define the type of parameters triggering the payout and the overall scope of intensity and frequency of the events covered by the mechanism.

The payout influences the ability of the recipient, such as a household and government, to cope with the short-, medium- and long-term consequences of the hazard. Some insurance mechanisms do not cover 100% of the insured value. Sometimes, parametric insurers calculate payout ex ante disaster and do not use ex-post assessments; therefore, there can be a difference between the ex ante loss model and the ex-post reality. Other payout-related factors include (1) payout trigger, meaning that the hazard event might be just under the trigger level and (2) the excess, deductible or copay (Suarez & Linnerooth-Bayer 2011).

The reserve or reinsurance enables the insurance mechanism to cover exceptionally high claims. Without a safe reserve or reinsurance mechanism, the insurance company can go bankrupt, leaving policyholders without sufficient cover. Several insurance companies went bankrupt after Hurricane Andrew hit Florida in 1992 (Kunreuther 1996). To ensure the financial stability and reliability of any insurance mechanisms set up in the Pacific SIDS, the reserve and the reinsurance should be sufficiently funded and accessible while being safely managed or invested.

Policy measures to increase the DRR incentives would help limit the growth of insurance mechanism costs by reducing vulnerability across the Pacific SIDS. If policy holders adopt DRR measures, then incentives might include lower premiums, lower copays or deductibles, and higher percentages or maxima of coverage (Crichton 2008; Kunreuther 1996). The contractual security ensures that the policyholder or the person transferring the risk to another entity is entitled to claim a payout or support. The scope of the coverage of the insurance mechanism is multi-faceted, referring to the types of assets the mechanism covers and the frequency, magnitude or intensity of events in the mechanism to trigger the coverage.

### Public insurance mechanisms

Using the six criteria described above, Table 1 compares the public insurance mechanisms.

**TABLE 1:** Comparing public insurance mechanisms to be considered for Pacific Small Island Developing States.

Insurance name → Insurance criteria ↓	Multi-country pool: CCRIF – Caribbean	Catastrophe-linked securities: MCB – Mexico	Public–private insurance: TCIP – Turkey	Post-disaster assistance
Premium – Calculation	Depends on the amount of coverage that the country wants, the attachment and exhaustion point of that coverage, and the risk profile of the country.	Premium of 4% + a further rate depending on the risk rating.	Risk zone and construction quality (the tariff) multiplied by the number of square metres of the house insured and by the unit square metre cost (sum insured).	There is no premium.
Premium – Cost	$21 838 512 for the 16 countries.	From 10.25% for the 3 different cyclone zones to 11.25% for the 3 different earthquake zones.	Average $62 per homeowner (depending on location and construction type).	There is no premium.
Payout – Trigger	Parametric: Richter scale or atmospheric pressure.	Parametric: Richter scale or atmospheric pressure.	Declaration of losses (claim).	Parametric: A disaster occurrence leading to offers of or requests for assistance.
Payout – Maximum	50% of the total estimated direct losses up to a sum between $1 and $104 million.	In 2006, for $160 million bond, maximum $450 million.	The maximum sum insured is approximately $92 000 (as of January 2009, because it is subject to the exchange rate) per owner.	None, but examples show that the pledges of assistance are not always fulfilled.
Reserve	$78.6 million (in 2009) from donors and loans.	No reserve.	$180 million (overall claim supported by the government).	The reserve is effectively as much as the donors could afford. That covers individual remittances, official aid, and private sector contributions.
Reinsurance	$132.5 million (private companies).	$290 million (managed by Swiss Re and Goldman Sachs Group).	Up to $1.5 billion.	There is no reinsurance *per se*.
DRR incentives	In development.	None.	Price incentive for dwellings in steel and reinforced concrete frame. The premium rates for dwellings in masonry and other structures are 175% – 250% above the premium rate of a dwelling built in steel and concrete.	Many humanitarian relief agencies integrate DRR measures into their relief operations. Donors could make DRR now a pre-condition for providing disaster relief later, but no examples of that were found.
Contractual security	CCRIF and the beneficiary countries enter a contract.	The bondholder and the beneficiary country enter a contract as well as the investors and bondholder.	The policyholder and TCIP enter a contract.	No legal or contractual relations.
Scope of the coverage – Intensity	Events in which the intensity crosses a contractually defined physical threshold.	Events in which the intensity crosses a contractually defined physical threshold.	The mechanism is based on claims, not intensity.	At the discretion of external donors.
Scope of the coverage – Sectors	Government budget for a post-disaster liquidity gap.	Government budget for reconstruction.	For private households.	At the discretion of external donors.

Source: CCRIF (2010); Ferris (2011); GFDRR and World Bank (2011a, 2011b); Mahul and Cummins (2009); Maynard (2008); McGee and Rodriguez (2009); Michel-Kerjan *et al*. (2011); TCIP (2011); UNECLAC (2010); World Bank (2008, 2010a).

DRR, disaster risk reduction; CCRIF, Caribbean Catastrophe Risk Insurance Facility; MCB, Mexico Catastrophe Bond; TCIP, Turkish Catastrophe Insurance Pool.

The four mechanisms display a large diversity, with wide differences within each category. For instance, for DRR incentives, CCRIF’s are under development, MCB does not have any, TCIP’s are structural and disaster aid has all forms of DRR depending on the donor and mechanism. CCRIF, MCB and TCIP rely on contractual relations between the insurance mechanisms and the beneficiaries, whereas there are neither legal nor contractual relations in the case of post-disaster assistance. The scope of the coverage diverges. MCB and CCRIF are parametric mechanisms insuring government budgets either for reconstruction (MCB) or for the liquidity gap post-disaster (CCRIF). Meanwhile, TCIP insures private households and payout is based on claims. The scope of the coverage for post-disaster assistance is at the discretion of the external donors and support.

Some similarities emerge with regard to the trigger for a payout because three of the mechanisms have a parametric trigger, but each using different parameters. CCRIF’s parametric trigger is particularly high and selective. For example, the countries are covered for damage caused by cyclonic winds, not necessarily for floods caused by the same cyclone.

### Private insurance mechanisms

Using the same six criteria described above, Table 2 compares the private insurance mechanisms.

**TABLE 2:** Comparing private insurance mechanisms to be considered for Pacific Small Island Developing States.

Insurance name → Insurance criteria ↓	Risk retention	Private individual policies: QBE – Vanuatu	Social safety net: HARITA – Ethiopia	Micro-insurance: RKBY – India
Premium – Calculation	There is no premium.	Depends on the type of construction, the risk profile of the area, and the limit of liability desired.	Depends on the risk of drought and on the area insured.	Percentage between 1.5% and 3.5% or actuarial rate (if lower) of the total sum insured. The percentage depends on the crop insured.
Premium – Cost	There is no premium.	From 0.555% to 0.971% of the sum insured (0.588% on average).	Average $12 in labour or money. For the government, $930 000, supported by USAID; a 13.1% premium rate.	For 1 hectare of paddy (a marginal farmer), the full premium is $8.87 (premium rates 2.5% and 3.55% for average yield coverage) compared to $593.71 for the value of the average yield. Premium subsidies included are 1.49% of the total sum insured.
Payout – Trigger	A disaster affecting the self-insured (e.g. household, business, or government).	After declaration of losses (a claim is made).	Parametric, drought or rainfall index at a station.	Parametric for listed hazards.
Payout – Maximum	Whatever the self-insured can afford.	Depends on the limit of liability desired and the excess.	Based on the distribution of rainfall observed since 1950, resulting in an average payout that is 6% of the total insured value.	Different levels of compensation: 60%, 80% and 90%, depending of the level of risk area.
Reserve	Depends on the maximum amount of funds that the self-insured can allocate.	Not public domain.	Not public domain.	The Calamity Relief Fund: Equal participation from the national government and the state (sub-national) government.
Reinsurance	Not applicable.	Not public domain.	2006/2007 Axa Re $7.1 million and 2010 Swiss Re $1.25 million.	International financial market.
DRR incentives	The self-insured has an incentive to try to limit the losses incurred. No monitoring exists to see whether or not DRR measures are affordable or are taken – or, if taken, are implemented fully and properly.	Only dwellings complying with the cyclone certificate delivered by a local engineer or an architect can be insured.	Tree planting, water harvesting, seed cleaning, and composting, all of which assist in maintaining post-disaster self-sufficiency. Trees can serve as a wind break and flood alleviator for lower category cyclones. If they are uprooted by winds or floods, then they can become dangerous debris.	None.
Contractual security	Absent.	The policyholder and the insurance company enter a contract.	The policyholder and the social safety net programme enter a contract.	The policyholder and the micro-insurance company enter a contract.
Scope of the coverage – Intensity	Limited to the self-insured’s capacity.	The mechanism is based on claims, not intensity.	Events in which the intensity crosses a contractually defined physical threshold.	Events in which the intensity crosses a contractually defined physical threshold.
Scope of the coverage – Sectors	Limited to the self-insured’s capacity.	For private households.	Agricultural households.	Agricultural households.

Source: Agriculture Insurance Company of India Limited (2010); Government of India (c. 2014); Integrated Regional Information Networks (2007); Mahul and Stutley (2010); McCabe (2009); Meze-Hausken, Patt & Fritz (2009); Mortimer (2011); Osgood (2010); Shorten *et al*. (2003).

DRR, disaster risk reduction; ; HARITA, Horn of Africa Risk Transfer for Adaptation; USAID, United States Agency for International Development; RKBY, Rashtriya Krishi Bima Yojana.

Risk retention is an outlier compared to the other three mechanisms because no other parties are involved. The ‘policyholder’ makes all decisions, within the limits of their own resources.

The other three mechanisms have many similarities. Different types of insurance and safety net programmes are available for purchase, depending on the level of income and the compensation expected. The premium calculation method is similar, which is a function of the sum insured and the risk covered. They rely on a written or an oral contract between the policyholders and the insurance mechanism or social safety net. Entering a contract ensures that both parties meet their insurance obligations: the policyholder is entitled to receive a payout when the parameter is triggered, while the insurance company or social safety net is contractually entitled to receive the agreed premium, through work (HARITA) or cash (all three).

The triggers of the three mechanisms, still including risk retention, are different, and a major difference emerges in the amount of compensation that the insurance can offer. For droughts, in Ethiopia, the average compensation is 6% of the sum insured, whereas in India it fluctuates between 60% and 90% of the sum insured, depending on the service chosen by the customer and the customer’s location. The reserve and reinsurance approach is different for each mechanism. The DRR mechanisms also differ. For Vanuatu’s private individual policies, the DRR measures are structural; in Ethiopia, they are non-structural; and in India, they are not present. The example from Vanuatu appears to be solid DRR, but might end up being an obstacle to insurance for households that are unable to afford construction meeting the certificate standard. Also there might exist other reasons for avoiding such dwellings, such as preferring traditional building materials and buildings more suited to the tropical climate.

## Evaluating insurance mechanisms for the Pacific Small Island Developing States

### Average premium and country’s income

The different insurance mechanisms studied are implemented in countries with various stages of development, ranging from comparatively rich (such as Bahamas in CCRIF) to comparatively poor (such as India) – a diversity of affluence reflected across the Pacific SIDS. Despite the disparities and to improve the comparison amongst different mechanisms, the average premium paid for the coverage as found in the literature is divided by the gross national income (GNI) per capita, purchasing power parity (PPP) from the World Bank (2015), not including risk retention and post-disaster assistance, because they do not have premiums. QBE in Vanuatu yielded a ratio of 31%, while the other five mechanisms yielded a ratio of below 1%. The high ratio for Vanuatu is mirrored across many other Pacific SIDS, indicating that private insurance along the lines of QBE’s product would be generally unaffordable around the region. Furthermore, for the lowest bands of sum insured (between $6683 and $13 366), the average premium paid by the homeowners is $102.25 (Shorten *et al*. 2003), which is approximately 2 weeks’ income for the average Pacific islander.

This comparison, however, does not give a specific overview of the wealth of the population purchasing the insurance policy (only the average wealth of the population) nor does it account for income disparities within countries’ populations. Even with RKBY’s ratio well below 1%, it might still be unaffordable for many Indians. HARITA helps to overcome these barriers by permitting the premium to be paid through work rather than cash. As an element of comparison regarding the aggregate cost of premiums reported to the GDP per capita, insurance penetration averages at $2750 per capita (or 9% of the average GDP per capita) in countries labelled as ‘developed’ and $25 per capita (or 5% of the average GDP per capita) in other countries (Mills 2005).

### Level of compensation

Levels of compensation are provided in Tables 1 and 2. For payout compared to the damage cost, post-disaster assistance is hoped to be 100%, but is donor-dependent, while risk retention depends on the assets and capacities of the entity (e.g. government, household or business) retaining the risk. For two mechanisms – HARITA and MCB – this comparison cannot be made because the payout is not linked to the damage cost; instead, it depends both on liability purchased by the insured and on the excess or deductible.

Factors that limit the amount of insurance payout include maximum payout permitted, maximum sum insured, the excess and the location. Furthermore, in the case of the specific insurance companies, other exclusion clauses can decrease the payout. For example, for QBE in Vanuatu, the insurance policy does not cover storm damage from the sea or high tides (QBE Insurance Limited 2011), which is not helpful for properties damaged by a tropical cyclone’s storm surge. Such limitations can make an insurance mechanism irrelevant for many Pacific SIDS communities – such as in Tuvalu and atolls of PNG – where a significant amount of public and private assets are located close to the shoreline in low-lying areas with high tides frequently causing damage.

Also, disputes can arise regarding the insured’s claims of losses compared to the insurance’s calculation of losses. This can lead to payout delay, which can make the insurance almost irrelevant on remote islands where materials for rebuilding can take weeks to arrive; therefore, the materials need to be ordered and paid for as soon as possible.

CCRIF has been specifically developed to overcome the liquidity gap that occurs in the immediate aftermath of a disaster by not requiring verification of losses (Ghesquiere & Mahul 2007). The parametric trigger, however, means that no payout is available when the hazard is just below the parameter, even if extensive damage was experienced (Suarez & Linnerooth-Bayer 2011). Cyclones are a particular problem when rainfall, rather than wind speed related to atmospheric pressure, causes major damage. Some insurance mechanisms also limit the maximum sum insured, thereby limiting the payout.

### Reserve and reinsurance

The financial and political stability and reliability of an insurance mechanism depend on its ability to compensate exceptional losses without going bankrupt. Six mechanisms (excluding risk retention and post-disaster assistance) have developed a reserve or contracted reinsurance policy, but not all data are publicly available (Table 2). Nonetheless, how could the Pacific SIDS learn from these other mechanisms?

For TCIP and RKBY, the governments of Turkey and India, respectively, have the role of reserve for the overall claim and of reinsurer for the local governments. CCRIF has not taken that route because neither its member governments nor Caribbean supranational organisations would have enough assets – a similar situation for the Pacific which might need to follow CCRIF’s route in developing a reserve through donations and development banks. HARITA (Ethiopia) does not have a reserve, but receives reinsurance through Swiss Re as a ‘founding sponsor’ (Swiss Re 2011) in collaboration with Oxfam America and the World Food Programme, another possibility for the Pacific SIDS to pursue.

For MCB, Mexico’s government through the Natural Disaster Fund of the Government of Mexico (FONDEN) entered into an insurance contract with a local reinsurer (Agro Asemex), which has entered into a reinsurance contract with Swiss Re (Michel-Kerjan *et al*. 2011). MCB does not have a reserve. For QBE in Vanuatu, the reinsurance is through QBE’s reinsurer or through the reinsurers of the companies selling the policies (QBE Insurance Limited 2011). Finding a willing reinsurance, whether of one layer or of many layers, could serve the Pacific SIDS.

The reserve and the reinsurance have to be safely managed and sufficiently funded. Currently, risk probability calculations for the Pacific SIDS for tropical cyclones and droughts need to be refined to determine the probable maximum loss functions across multiple scenarios. Consequently, rough estimates with contingency would be needed to ensure that any Pacific SIDS insurance mechanism(s) could sustain expected losses. Existing insurance mechanisms tend to aim to sustain between a 1-in-250-year and a 1-in-1000-year loss (Mahul & Cummins 2009); however, the exact level would need to be determined, especially considering social and environmental changes that affect loss modelling and actual losses.

### Disaster risk reduction measures

Implementing insurance-linked DRR measures can significantly reduce the cost of disaster damage and losses (Crichton 2008). Out of the eight different insurance mechanisms studied (Tables 1 and 2), two mechanisms do not consider DRR measures and CCRIF is developing them. As such, any insurance mechanism implemented in the Pacific SIDS should have DRR incentives embedded from the beginning, rather than trying to include them afterwards.

TCIP has developed price incentives for the owners of dwellings built in steel and reinforced concrete, based on the earthquake hazard. By increasing the premium rate by 175% – 250% for the other structure types, TCIP gives a strong DRR incentive; however, monitoring and enforcement of such mechanisms are traditionally insufficient particularly because of corruption (Lewis 2003). Furthermore, construction in steel and reinforced concrete is not common in the Pacific SIDS for climate and cost reasons.

Also when considering tropical cyclones and droughts, the insurance mechanism would need to target DRR measures for those hazards specifically. For instance, for cyclones, certain construction materials could be the DRR incentive, but construction method should also be delineated, such as tying the roof to the walls and the walls to the foundation. For droughts, the DRR method might be specific types of crops or else implementing water conservation methods such as drip irrigation. Monitoring and enforcement of the DRR measures would be needed, recognising that the insurance mechanism moves increasingly towards a governance mechanism by directing and policing livelihoods. Yet many Pacific SIDS DRR measures for tropical cyclones and droughts are part of the islanders’ traditional knowledge (e.g. Boyle 1992; Kelman *et al*. 2011; Lewis 1999; Reardon 1992), which has been eroded because of modernism. An insurance mechanism could have a role in combining traditional and modern knowledge forms for DRR, using techniques pioneered in the Pacific (e.g. Daly *et al*. 2010; Mercer *et al*. 2010).

Care may be needed to ensure that risk retention does not become the default mechanism. While risk retention provides long-term incentives to implement DRR measures, short-term lack of affordability and knowledge can preclude action. This concern is particularly relevant for the Pacific SIDS because of the low GDP per capita and the indebtedness of governments inhibiting DRR investment. Without financial and technical assistance, it is unlikely that self-implemented DRR measures would be popular – again highlighting the aid dependency of the Pacific SIDS which can, in turn, undermine traditional DRR and coping mechanisms.

### Contractual security

Both at the household and government levels, policyholders and beneficiaries need to have confidence that they can contractually rely on the mechanism to receive post-disaster indemnification and that pledges, including contracts with insurers, are matched by action. For aid, in the absence of legal or contractual relations with the donor countries, the recipient country cannot oblige donors to disburse pledged funds.

Risk retention removes reliance on others, but generates its own problems. Risk retention and the absence of secured compensation can trap households, businesses and governments into poverty if they are repeatedly hit by tropical cyclones and droughts without having enacted DRR measures. The majority of the Pacific SIDS already have high indebtedness. For example, Samoa and Palau each had debt amounting to about 50% of the GDP (in 2011), while Nauru had a debt three times higher than its GDP. Loans simply perpetuate this cycle, especially if they focus on disaster recovery rather than DRR.

To break the cycle of debt, aid dependency and disaster, the Pacific SIDS need a prompt and reliable mechanism. Supporting DRR measures would be needed and paying for full costs would assist. Unambiguous contractual security could assist with both processes.

### Scope of the insurance mechanisms

Scope is defined by two underlying mechanism elements: the event intensity – which could be defined by hazard, vulnerability, or risk – and the sectors covered. Amongst the eight mechanisms, only TCIP indemnifies policyholders based on losses claimed. The other mechanisms use a parametric trigger. Consequently, all losses and damage incurred by events below the physical parametric trigger are not covered, effectively forcing risk retention as the default mechanism. In particular, MCB’s parametric trigger is set to indemnify for high-intensity, low-frequency events (GFDRR & World Bank 2011b). Setting high thresholds for parametric triggers for Pacific SIDS is unsuitable because the small size of the communities means that even a small tropical cyclone or drought can have major consequences if vulnerability has not been redressed (Kelman *et al*. 2011; Lewis 1999).

Regarding sectors covered, HARITA and RKBY cover only agricultural losses, TCIP and QBE cover property and asset losses, CCRIF and MCB effectively cover a government’s budget that could be used across sectors, and risk retention and aid cover any sector(s) selected. While droughts in Pacific SIDS primarily affect agriculture and freshwater, livelihoods are so interconnected in small communities that most sectors would be affected – as they would be with most cyclones. Consequently, having a multi-sectoral insurance mechanism, especially supporting DRR measures across all sectors, would be preferred for Pacific SIDS.

## Discussion: Comparing insurance mechanisms for Pacific Small Island Developing States

### Radar charts methodology

The insurance mechanism elements analysed in the previous section are not independent; together, they indicate the reliability, safety, cost and performance of an insurance mechanism, adding up to suitability. For comparing insurance mechanisms, according to such parameters, radar charts (nested polygons with parameter values at each vertex) are used regularly (Clarke & Garside 1997; Schmid, Schütz & Speckesser 2003). Here, using the radar charts provides a visual decision support tool to help Pacific SIDS define their own choice based on a particular focus, for instance, if they prefer a low premium or high payout. The parameters analysed here are:

#### Affordability

Four categories are taken into account: the mechanism is free (no premium has to be paid); very low (above zero up to 0.01% of the GNI, PPP), low (above 0.01% up to 1.0%), high (above 1% up to 30%) and very high (above 30%)

#### Compensation

Five levels are considered: the mechanism compensates up to 100% of the losses claimed; it compensates the full losses until a defined amount; the mechanism compensates a high percentage of the losses; the mechanism compensates a low percentage of the losses; and the mechanism compensates only a pre-defined amount including a maximum amount.

#### Disaster risk reduction incentive

This is ordered according to the strength of the DRR measures being included in the insurance mechanism: compulsory DRR, price-incentivised DRR, DRR incentives without monitoring, DRR measures under development and no DRR.

#### Contractual or legal security

For this item, two options are possible: yes, the parties enter a contractual agreement and no, the parties do not enter a contractual agreement, although forms of soft contracts might be feasible such as a soft contract, such an agreement or memorandum of understanding which the parties accept might not be legally binding or legally enforceable.

#### Scope of the mechanism

The mechanisms are analysed according to two factors: whether the risks covered are limited by an index and whether the cover is mono- or multi-sectoral.

For a cross-mechanism comparison, reinsurance and reserve are not particularly helpful because of the lack of data. The main disadvantage with radar charts is that the distance between nested polygons does not represent true distances between values at the vertices because they are effectively ordinal rankings rather than cardinal numbers; the radar charts are very much a visual tool providing many comparative data to support, not make, decisions.

### Public insurance mechanisms

**FIGURE 1:**
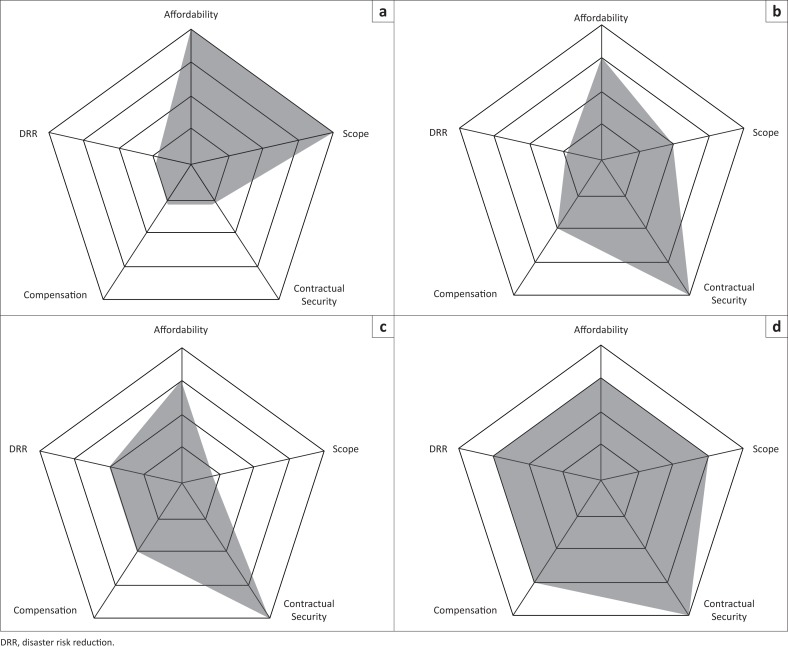
Radar charts for public insurance mechanisms. (a) Post-disaster assistance, (b) catastrophe-linked securities (Mexico Catastrophe Bond), (c) multi-country pool insurance (Caribbean Catastrophe Risk Insurance Facility) and (d) compulsory insurance (Turkish Catastrophe Insurance Pool).

Figure 1 is the radar chart for the four types of public insurance mechanisms considered here, demonstrating clear differences. For compensation, the mechanisms divide into two clear categories, high compensation (from TCIP) and low compensation (from MCB and CCRIF). Post-disaster assistance is hard to determine exactly because humanitarian aid amounts can never be projected in advance and is not always connected with the actual losses – in addition to wide gaps between (1) aid pledged and aid received and (2) aid arriving in a country and aid reaching disaster-affected people (e.g. Ferris 2011). Consequently, because of its unreliability, post-disaster assistance is ranked low in terms of compensation. Another difference highlighted is DRR incentives because the four mechanisms have four different incentive approaches. Based on Figure 1, post-disaster assistance might appear to be the most advantageous public insurance mechanism; given that the premium is non-existent, the payout is supposed to be high; and some donor countries impose DRR measures. Key disadvantages, namely dependency and that pledges might not be met, do not come through on the radar charts.

Examining the similarities, all four mechanisms are affordable. TCIP does that deliberately because it is compulsory. For the other mechanisms, the low relative cost of the premiums is helpful for the relatively high poverty levels in Pacific SIDS. Yet low premiums lead to relatively low compensation for CCRIF and MCB, which is unhelpful for breaking the poverty and dependency cycles in which many Pacific SIDS remain trapped. Consequently, any public insurance mechanism for the Pacific SIDS might need to avoid CCRIF’s and MCB’s pattern because then reliance on post-disaster aid is likely to remain attractive.

For the Pacific SIDS, could mechanisms such as CCRIF and MCB be improved in terms of compensation without reducing affordability (see also McGee *et al*. 2014)? TCIP provides reasonable compensation, partly because of the size of the risk pool, in that Turkey has a large population and the insurance is compulsory. Mexico’s population is much larger than Turkey’s, but MCB is not compulsory. The Pacific SIDS might therefore wish to consider a compulsory insurance mechanism to achieve the size of the risk pool needed for high affordability and high compensation. If PNG is not involved, though, it is questionable whether or not the risk pool could ever be large enough amongst all the other Pacific SIDS.

Given the Pacific SIDS’s ongoing regional cooperation for DRR, technical and management capability for the insurance mechanism could be built in one supranational institute, helping to lower costs. Additionally, for cyclones, a risk pool across the Pacific is likely to be successful because a single cyclone rarely affects more than a few countries. Droughts could be more problematic because most of the Pacific SIDS can be affected simultaneously.

Yet it is not clear that any premium level would be affordable for Pacific islanders, given the low rates of cash income and high rate of subsistence living. Emulating HARITA, a work-for-premium scheme might be sensible – but many Pacific cultures already have a deeply engrained sense of community and communal work, so it would be difficult and potentially highly disruptive to suddenly suggest that a specific component of community work goes towards disaster insurance.

### Private insurance mechanisms

**FIGURE 2:**
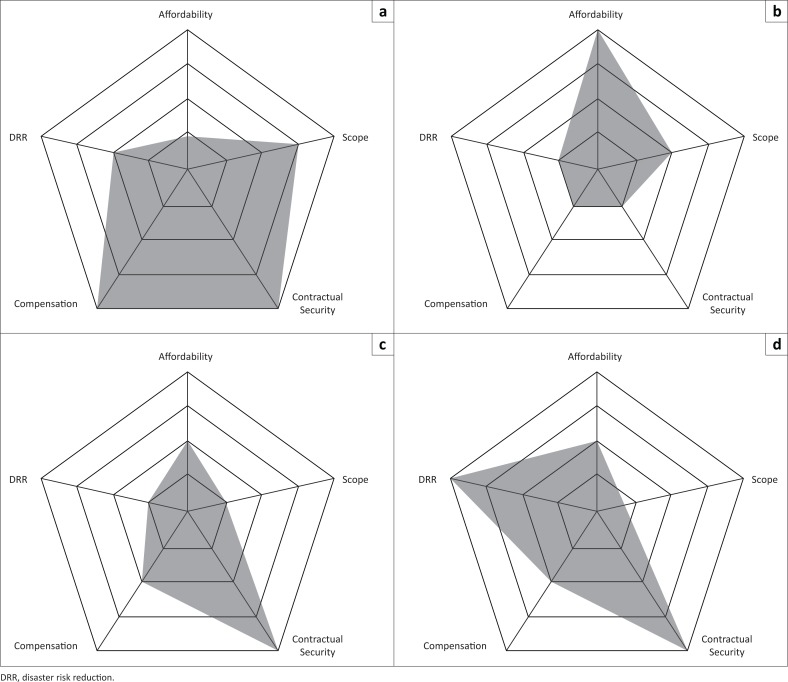
Radar charts for private insurance mechanisms. (a) Private individual policies (QBE Vanuatu), (b) risk retention, (c) micro insurance (Rashtriya Krishi Bima Yojana) and (d) social safety net (Horn of Africa Risk Transfer for Adaptation).

Figure 2 is the radar chart for the four types of private insurance mechanisms considered here. Apart from private individual policies, the insurance mechanisms are affordable with negligible premiums; however, the core part of RKBY’s premium is subsidised for small and marginal farmers at a 50% level (Agriculture Insurance Company of India Limited 2010). This approach might be appropriate for the Pacific SIDS to consider in order to encourage insurance uptake – provided that governments or donors can afford that level of subsidy. As discussed above, building on HARITA, a Pacific SIDS insurance mechanism with premiums paid by in-kind, community DRR-related work by the insured people could further boost local culture and identity.

The issue of the risk pool’s size for the Pacific SIDS was raised for public insurance mechanisms and emerges here too. For small numbers of customers, the transaction costs of private individual policies or of micro-insurance (McGuire 2000) could significantly increase the premium’s cost to a price that is unaffordable. Subsidising the Pacific SIDS’ governments with aid or loans to lower the premium cost could simply continue aid dependency or indebtedness. For example, RKBY in India relies mainly on the Indian government subsidising the premium and the reserve. The Pacific SIDS governments are not in a financial position to act similarly.

As with the public insurance mechanisms, drought is likely to be problematic regarding compensation to many people at the same time. Making the premiums high enough to create an adequate reserve reduces affordability. Instead, implementing or mandating drought risk reduction measures based on combining traditional and scientific knowledge (Mercer *et al*. 2010) would appear to be key for a reliable drought insurance policy. Reinsurance could be an option, especially considering that a drought reinsurance payout for the Pacific region would be small compared to the amount of compensation that most reinsurers deal with. Affordability of reinsurance depends on the premium.

## Conclusion

This article has explored possibilities for implementing insurance mechanisms in Pacific SIDS for droughts and tropical cyclones. The text has examined opportunities and constraints or limitations of a selection of eight existing insurance mechanisms over seven insurance categories. Limitations especially emerge regarding reliance on post-disaster assistance or risk retention since these mechanisms are not reliable. Additionally, private individual policies, through exclusion clauses and premium costs, are likely to be of limited value.

The other mechanisms depend on two principal elements for their implementation: (1) political will involving government decisions and (2) internal or external technical and/or financial assistance to set up a specific insurer – which could be for-profit, not-for-profit, a government, or a group of governments. Element (1) could emerge from element (2), especially given the Pacific SIDS’ long-term regional cooperation organisations which are trusted by the governments and which have a long track record of DRR around the Pacific. Similarly, element (2) could emerge from element (1). If the governments make a decision that the Pacific SIDS need insurance, then they could determine which approach to select and where to find the needed external assistance.

Deliberately, this article has not provided a specific recommendation for an insurance mechanism to use for Pacific SIDS. Instead, the positives and negatives of different approaches have been discussed so that decision-makers can seek to balance the opportunities and the challenges. But who are the decision-makers? Who will decide that the Pacific SIDS should or should not have one or more disaster insurance mechanisms, possibly starting with tropical cyclones and droughts or possibly starting with other, or all, hazards?

If the decision is made to implement disaster insurance for the Pacific SIDS, who has the power and authority to select and implement the mechanism(s)? Individuals who can afford it can make decisions for their own insurance. The governments of Pacific SIDS have the power to make such decisions for all their populations, but could use advice from their supranational organisations and also have the power to permit or restrict the private sector to offer insurance (and reinsurance) services.

Alternatively, insurance-related decisions might be made by donors, such as those with the reserve, who direct the SIDS’ governments regarding insurance-related decisions – which might lead to the status quo. Given the toll which droughts, tropical cyclones and other hazards have long taken on Pacific SIDS’ lives and livelihoods, it would assist if an informed decisions were made, instead of lack of change through inertia.

## References

[CIT0001] ADRA Australia, 2010, ‘Fiji cyclone: ADRA provides emergency food supplies’, viewed 09 August from http://reliefweb.int/report/fiji/fiji-cyclone-adra-provides-emergency-food-supplies

[CIT0002] Agriculture Insurance Company of India Limited, 2010, ‘National Agricultural Insurance Scheme – Salient features – Levels of indemnity and threshold yield’, viewed 18 August 2015, from http://www.aicofindia.com/AICEng/Pages/Product_Profile/Present_NAIS_Features_P7.aspx

[CIT0003] American Meteorological Society, 1997, ‘Meteorological drought – Policy statement’, *Bulletin of the American Meteorological Society* 78, 847–849.

[CIT0004] Australian Bureau of Meteorology, 2015, ‘Tropical cyclones’, viewed 26 July 2015, from http://www.bom.gov.au/climate/environ/cyclones.shtml

[CIT0005] Boyle C., 1992, ‘Disaster resistant construction for small dwellings in Solomon Islands’, in Aysan Y. & Davis I. (eds.), *Disasters and the small dwelling: Perspectives for the UN IDNDR*, pp. 183–188, James & James, London, UK.

[CIT0006] CCRIF, 2010, ‘A guide to understanding CCRIF’, CCRIF, Georgetown, p. 20.

[CIT0007] Clarke A. & Garside J., 1997, ‘The development of a best practice model for change management’, *European Management Journal* 15(5), 537–545. http://dx.doi.org/10.1016/S0263-2373(97)00033-9

[CIT0008] Crichton D., 2008, ‘Role of insurance in reducing flood risk’, *Geneva Papers on Risk and Insurance* 3, 117–132. http://dx.doi.org/10.1057/palgrave.gpp.2510151

[CIT0009] Cummins J.D. & Trainar P., 2009, ‘Securization, insurance, and reinsurance’, *The Journal of Risk and Insurance* 76(3), 463–492. http://dx.doi.org/10.1111/j.1539-6975.2009.01319.x

[CIT0010] Daly M., Poutasi N., Nelson F. & Kohlhase J., 2010, ‘Reducing the climate vulnerability of coastal communities in Samoa’, *Journal of International Development* 22(2), 265–282. http://dx.doi.org/10.1002/jid.1678

[CIT0011] Ferris E.G., 2011, *The politics of protection: The limits of humanitarian action*, The Brookings Institute, Washington, DC.

[CIT0012] French Embassy in Australia, 2011, ‘FRANZ meeting – Sydney’, viewed 14 December 2015, from http://www.ambafrance-au.org/spip.php?article1641.RSLS

[CIT0013] GFDRR, World Bank, 2011a, ‘Turkish Catastrophe Insurance Pool – Providing affordable earthquake risk insurance’, GFDRR (Global Facility for Disaster Reduction and Recovery), Washington, D.C., pp. 1–2.

[CIT0014] GFDRR, World Bank, 2011b, ‘Mexico MultiCat Bond – Transferring catastrophe risk to the capital markets’, GFDRR (Global Facility for Disaster Reduction and Recovery), Washington, D.C., p. 1–2.

[CIT0015] Ghesquiere F. & Mahul O., 2007, *Sovereign natural disaster insurance for developing countries: A paradigm shift in catastrophe risk financing*, Working paper, The World Bank, Washington, DC.

[CIT0016] Glantz M.H. & Katz R.W., 1977, ‘When is a drought a drought?’, *Nature* 267, 192–193. http://dx.doi.org/10.1038/267192a0

[CIT0017] Government of Fiji, 2010, ‘Colgate Palmolive assists health’, viewed 19 November 2015, from http://reliefweb.int/report/fiji/fiji-colgate-palmolive-assists-health

[CIT0018] Government of India, c, 2014, *Revised: National Agricultural Insurance Scheme (NAIS) (Rashtriya Krishi Bima Yojana-RKBY)*, Department of Agriculture & Cooperation and Farmers Welfare, Ministry of Agriculture and Farmers Welfare, Government of India, Delhi, India.

[CIT0019] Heim R.R. Jr. 2002, ‘A review of twentieth-century drought indices used in the United States’, *Bulletin of the American Meteorological Society* 83, 1149–1165. http://dx.doi.org/10.1175/1520-0477(2002)083%3C1149:AROTDI%3E2.3.CO;2

[CIT0020] Integrated Regional Information Networks, 2007, ‘Ethiopia: Drought insurance extended to 6.7 million people’, viewed 06 October 2015, from http://reliefweb.int/node/252106

[CIT0021] Kelman I., Lewis J., Gaillard J.C. & Mercer J., 2011, ‘Participatory action research for dealing with disasters on islands’, *Island Studies Journal* 6(1), 59–86.

[CIT0022] Kunreuther H., 1996, ‘Mitigating disaster losses through insurance’, *Journal of Risk and Uncertainty* 12(2–3), 171–187. http://dx.doi.org/10.1007/BF00055792

[CIT0023] Le De L., Gaillard J.C., Friesen W. & Matautia Smith F., 2015, ‘Remittances in the face of disasters: A case study of rural Samoa’, *Environment, Development and Sustainability* 17(3), 653–672. http://dx.doi.org/10.1007/s10668-014-9559-0

[CIT0024] Lewis J., 1999, *Development in disaster-prone places: Studies of vulnerability*, Intermediate Technology Publications, London.

[CIT0025] Lewis J., 2003, ‘Housing construction in earthquake-prone places: Perspectives, priorities and projections for development’, *The Australian Journal of Emergency Management* 18(2), 35–44.

[CIT0026] Linnerooth-Bayer J. & Mechler R., 2006, ‘Insurance for assisting adaptation to climate change in developing countries: A proposed strategy’, *Climate Policy* 6, 1–16. http://dx.doi.org/10.1080/14693062.2006.9685628

[CIT0027] Mahul O. & Cummins J.D., 2009, *Catastrophe risk financing in developing countries – Principles for public intervention*, The World Bank, Washington, DC.

[CIT0028] Mahul O. & Stutley C.J., 2010, *Government support to agricultural insurance – Challenges and options for the developing countries*, World Bank Publications, Washington, DC.

[CIT0029] Maynard T., 2008, ‘Climate change: Impacts on insurers and how they can help with adaptation and mitigation’, *The Geneva papers on risk and insurance – Issues and practice* 33, 140–146. http://dx.doi.org/10.1057/palgrave.gpp.2510154

[CIT0030] McCabe C., 2009, ‘Weather insurance offers Ethiopian farmers hope-despite drought’, viewed 14 December 2015, from http://www.oxfamamerica.org/explore/stories/weather-insurance-offers-ethiopian-farmers-hopedespite-drought

[CIT0031] McGee J., Phelan L. & Wenta J., 2014, ‘Writing the fine print: Developing regional insurance for climate change adaptation in the Pacific’, *Melbourne Journal of International Law* 15(2), 444–472.

[CIT0032] McGee J. & Rodriguez C.M., 2009, ‘Mexico issues catastrophe bonds through world bank’, viewed 10 March 2013, from http://www.bloomberg.com/apps/news?pid=newsarchive&sid=aHNdLXzshPxk

[CIT0033] McGuire P.B., 2000, ‘South Asian economic models for the Pacific? The case of microfinance’, *Pacific Economic Bulletin* 15(1), 168–172.

[CIT0034] Mercer J., Kelman I., Taranis L. & Suchet-Pearson S., 2010, ‘Framework for integrating indigenous and scientific knowledge for disaster risk reduction’, *Disasters* 34(1), 214–239. http://dx.doi.org/10.1111/j.1467-7717.2009.01126.x 19793324 10.1111/j.1467-7717.2009.01126.x

[CIT0035] Meze-Hausken E., Patt E. & Fritz S., 2009, ‘Reducing climate risk for micro-insurance providers in Africa: A case study of Ethiopia’, *Global Environmental Change* 19, 66–73. http://dx.doi.org/10.1016/j.gloenvcha.2008.09.001

[CIT0036] Michel-Kerjan E., Zelenko I., Cardenas V. & Turgel D., 2011, *Catastrophe financing for governments: Learning from the 2009–2012 MultiCat program in Mexico*, OECD Working Papers on Finance, Insurance and Private Pensions, No. 9, OECD Publishing, Paris, France.

[CIT0037] Mills E., 2005, ‘Insurance in a climate of change’, *Science* 309, 1040–1044. http://dx.doi.org/10.1126/science.1112121 16099975 10.1126/science.1112121

[CIT0038] Mortimer S., 2011, ‘Swiss Re joins Ethiopian micro-insurance project’, viewed 11 September 2015, from http://www.reuters.com/article/2011/06/10/microinsurance-swiss-re-idUSLDE7591EY20110610

[CIT0039] Osgood D., 2010, *HARITA IRI report to Oxfam America. Final report for IRI MIEL Planning & Technical Support for HARITA Micro-Insurance Pilot USA 536/09*, IRI Columbia, New York.

[CIT0040] QBE Insurance Limited, 2011, ‘Home cover – Insurance product disclosure statement and policy wording’, viewed 18 December 2015, from http://www.qbe.com.au/content/idcplg?IdcService=GET_FILE&dID=20747&dDocName=PRODCT035268

[CIT0041] Reardon G., 1992, ‘Wind effects on the Tongan “Hurricane House”’, in Aysan Y. & Davis I. (eds.), *Disasters and the small dwelling: Perspectives for the UN IDNDR*, pp. 175–182, James & James, London, UK.

[CIT0042] Reliefweb, 2004, ‘New Zealand offers Niue 20 million dollar aid’, viewed 09 August 2015, from http://reliefweb.int/node/411023

[CIT0043] Schmid G., Schütz H. & Speckesser S., 2003, ‘Broadening the scope of benchmarking: Radar charts and employment systems’, *Labour* 13(4), 879–899. http://dx.doi.org/10.1111/1467-9914.00119

[CIT0044] Shorten G.G., Goosby S., Granger K., Lindsay K Naidu P., Oliver S., 2003, *Catastrophe insurance pilot study, Port Vila, Vanuatu: Developing risk-management options for disasters in the Pacific Region*, SOPAC Joint Contribution Report 147, SOPAC (Secretariat of the Applied Geosciences Commission), Suva, Fiji.

[CIT0045] SOPAC, 2005, *Economic impact of natural disaster on development in the Pacific*, SOPAC (Secretariat of the Applied Geosciences Commission), Suva, Fiji.

[CIT0046] SPC, 2009, ‘Niue Statistics – National Accounts 2004’, viewed 01 October 2015, from http://www.spc.int/prism/niue/index.php/economic/national-accounts

[CIT0047] Suarez P. & Linnerooth-Bayer J., 2011, ‘Insurance-related instruments for disaster risk reduction’, in Maskrey A. (ed.), *Global assessment report on disaster risk reduction*, UNISDR, Geneva.

[CIT0048] Swiss Re, 2011, ‘Innovative weather insurance for farmers in Ethiopia is gaining momentum’, viewed 14 November 2015, from http://www.swissre.com/rethinking/food_security/Innovative_weather_insurance_for_farmers_in_Ethiopia_is_gaining_momentum.html

[CIT0049] TCIP, 2011, ‘Turkish Catastrophe Insurance Pool’, viewed 16 December 2015, from http://www.tcip.gov.tr

[CIT0050] Tutangata T. & Power M., 2002, ‘The regional scale of ocean governance regional cooperation in the Pacific Islands’, *Ocean and Coastal Management* 45, 873–884. http://dx.doi.org/10.1016/S0964-5691(02)00111-4

[CIT0051] UNDHA, 1990, ‘Cyclone Ofa February 1990 UNDRO Situation Reports 1–8, February 1990’, UNDHA (United Nations Department of Humanitarian Affairs), viewed 22 August 2015, from http://reliefweb.int/node/406868

[CIT0052] UNECLAC, 2010, ‘CCRIF and ECLAC create partnership for disaster risk reduction’, UNECLAC (United Nations Economic Commission for Latin America and the Caribbean), viewed 08 September 2015, from http://reliefweb.int/node/345932

[CIT0053] White I., Falkland T. & Scott D., 1999, *Droughts in small coral Islands: Case study, South Tarawa, Kiribati*, UNESCO (United Nations Educational, Scientific and Cultural Organization), Paris.

[CIT0054] World Bank, 2004, *Technical annex for a proposed grant in the amount of SDR 1.6 million and a proposed credit in the amount of SDR 1.4 million to the Independent State of Samoa for a cyclone emergency recovery project*, World Bank, Washington, DC.

[CIT0055] World Bank, 2006, *Adapting to natural hazards in the Pacific Islands region*, World Bank, Washington, DC.

[CIT0056] World Bank, 2008, *The CCRIF: Providing immediate funding after natural disasters*, World Bank, Washington, DC.

[CIT0057] World Bank, 2009, *IDA at work: Building cyclone resilient villages in Samoa*, World Bank, Washington, DC, viewed 14 December 2015, from http://web.worldbank.org/WBSITE/EXTERNAL/EXTABOUTUS/IDA/0,,print:Y~isCURL:Y~contentMDK:22300983~menuPK:3266877~pagePK:51236175~piPK:437394~theSitePK:73154,00.html

[CIT0058] World Bank, 2010a, *A review of CCRIF’s operations after its second season*, World Bank, Washington, DC.

[CIT0059] World Bank, 2010b, *World Bank to assist with tsunami reconstruction in Samoa*, World Bank, Washington, DC, viewed 14 December 2015, from http://web.worldbank.org/WBSITE/EXTERNAL/NEWS/0,,contentMDK:22752712~pagePK:64257043~piPK:437376~theSitePK:4607,00.html?cid=3001_3

[CIT0060] World Bank, 2015, ‘GDP per capita (current US$)’, viewed 14 December 2015, from http://data.worldbank.org/indicator/NY.GDP.PCAP.CD

